# DNA Methylation Dynamics During the Differentiation of Retinal Progenitor Cells Into Retinal Neurons Reveal a Role for the DNA Demethylation Pathway

**DOI:** 10.3389/fnmol.2019.00182

**Published:** 2019-07-24

**Authors:** Galina Dvoriantchikova, Rajeev J. Seemungal, Dmitry Ivanov

**Affiliations:** ^1^Department of Ophthalmology, Bascom Palmer Eye Institute, University of Miami Miller School of Medicine, Miami, FL, United States; ^2^Department of Microbiology and Immunology, University of Miami Miller School of Medicine, Miami, FL, United States

**Keywords:** retina, development, DNA methylation, retinal progenitor cells, retinal neurons, whole genome bisulfite sequencing

## Abstract

To evaluate the contribution of the DNA methylation and DNA demethylation pathways in retinal development, we studied DNA methylation in retinal progenitor cells (RPCs) and retinal neurons using a combination of whole genome bisulfite sequencing (WGBS) data obtained in our study and WGBS data collected from previous studies. The data was analyzed using Hidden Markov Model- and change point-based methods to identify methylome states in different segments of the studied genomes following genome annotation. We found that promoters of rod and cone phototransduction genes and rod photoreceptor genes, but not genes required for the development and function of other retinal phenotypes, were highly methylated in DNA isolated from human and murine fetal retinas (which mostly contain RPCs) and postnatal murine RPCs. While these highly methylated genomic regions were inherited by non-photoreceptor phenotypes during RPC differentiation, the methylation of these promoters was significantly reduced during RPC differentiation into photoreceptors and accompanied by increased expression of these genes. Our analysis of DNA methylation during embryogenesis revealed low methylation levels in genomic regions containing photoreceptor genes at the inner cell mass stage, but a sharp increase in methylation at the epiblast stage, which remained the same later on (except for DNA demethylation in photoreceptors). Thus, our data suggest that the DNA demethylation pathway is required for photoreceptor phenotypes in the developing retina. Meanwhile, the role of the DNA methylation and DNA demethylation pathways during RPC differentiation into non-photoreceptor retinal phenotypes might be less important.

## Introduction

While significant progress has been made in the understanding of signaling cascades that regulate retinal development, the role of epigenetic mechanisms has been explored to a lesser degree (Xiang, 2013; Corso-Díaz et al., 2018). DNA methylation creates epigenetic barriers that, while guiding and restricting differentiation of stem cells or progenitors into different cell types during development, also prevent regression into an undifferentiated state (Corso-Díaz et al., 2018). During development, patterns of methylated cytosines are established by the *de novo* methyltransferases Dnmt3a and Dnmt3b, and are subsequently preserved through cell divisions by Dnmt1, leading to inheritance of these patterns in descendants of stem cells or progenitors (Corso-Díaz et al., 2018). Emerging evidence suggests the equally important role of DNA demethylation in stem cell or progenitor differentiation into mature cell phenotypes (Koh and Rao, 2013; Huang and Rao, 2014). DNA demethylation can be carried out passively by not methylating (*via* Dnmt1) the newly synthesized strand after DNA replication, or actively by the DNA demethylation pathway, which includes the Ten-Eleven Translocation (TET) protein family (Koh and Rao, 2013; Huang and Rao, 2014). Thus, both the DNA methylation and DNA demethylation pathways are vital during cellular differentiation. Balance between DNA methylation and DNA demethylation during development is critical for cell differentiation and generation of normal tissue.

The role of the DNA methylation and DNA demethylation pathways during retinal development was investigated in different species, but no consistency was observed (Corso-Díaz et al., 2018). Triple Dnmt1, Dnmt3a, and Dnmt3b knockout mice showed reduced expression of phototransduction genes and lacked photoreceptor outer segments, which was followed by photoreceptor death (Singh et al., 2017). DNA methylation, tested using immunohistochemistry in these knockouts, was reduced in photoreceptor and retinal ganglion cell (RGC) nuclei. Meanwhile, knockouts of the TET protein family showed a similar phenotype. The many photoreceptor and RGC precursors in zebrafish Tet2/Tet3 double knockouts fail to differentiate into mature neurons (Seritrakul and Gross, 2017). The few photoreceptors that are still able to differentiate cannot form outer segments (Seritrakul and Gross, 2017). Tet3 demethylase controls eye development in *Xenopus* by promoting the expression of key developmental genes (Xu et al., 2012). The role of the DNA demethylation pathway in photoreceptor differentiation was also proposed in mice (Perera et al., 2015). Thus, regarding these studies, inactivation of two opposite pathways based on function (DNA methylation and DNA demethylation), leads to the same pathological phenotype in the developing retina; which is contradictory and requires clarification. The best approach to clarify these observations is to study the dynamics of DNA methylation in retinal progenitor cells (RPCs) and retinal cell types at single nucleotide resolution, since this reflects the activity of the DNA methylation and DNA demethylation pathways during retinal development. Changes in methylation of genes controlling retinal neuron phenotypes can show the contribution of the DNA methylation and DNA demethylation pathways during the development of these neurons. Recently, DNA methylation changes in a group of photoreceptor genes were investigated in developing rod photoreceptors using the reduced representation bisulfite sequencing (RRBS) approach (Kim et al., 2016). The authors found high levels of DNA methylation in promoters of a subset of rod-specific genes at the precursor stage, yet methylation levels in the same promoters were very low in mature rods (Kim et al., 2016). However, since the RRBS approach just covers up to 4 million CpG sites, mostly in CpG islands, many promoters and genes were not covered in the analysis. In our study, we collected data on the methylomes of RPCs and retinal cell types to study changes in DNA methylation during RPC differentiation into these retinal phenotypes. We used the whole genome bisulfite sequencing (WGBS) approach to eliminate the restrictions of RRBS and study the genomes of these retinal phenotypes with single nucleotide resolution.

## Materials and Methods

### Animals

All experiments were performed in compliance with the National Institutes of Health (NIH) Guide for the Care and Use of Laboratory Animals, the Association for Research in Vision and Ophthalmology (ARVO) statement for use of animals in ophthalmic and vision research, and the University of Miami Institutional Animal Care and Use Committee’s (IACUC) approved protocol. C57BL/6 J (stock number 000664) mice were obtained from the Jackson Laboratory (Bar Harbor, Maine, ME, United States). Mice were housed under standard conditions of temperature and humidity, with a 12-h light to dark cycle and free access to food and water.

### Isolation of RPCs (Notch1+ Cells) and RGCs

To isolate Notch1+ cells, we used the protocol for immunomagnetic separation described in Dvoriantchikova et al. (2015). Briefly, retinas were incubated in papain solution (16.5 U/mL; Worthington Biochemical Corp., Lakewood, NJ, USA) for 30 min to obtain the cell suspension. The retinal cell suspension was then incubated with monoclonal biotin conjugated anti-Notch1 mouse antibodies (130-096-557, Miltenyi Biotec, Auburn, CA, USA) followed by incubation with the anti-biotin microbeads (130-090-485, Miltenyi Biotec, Auburn, CA, USA). The cells were washed and used for magnetic separation of the Notch1+ cells on a pre-equilibrated column in the presence of a magnetic field according to the manufacturer’s protocol (Miltenyi Biotech, Auburn, CA, USA). To isolate RGCs, we used the two-step immunopanning protocol described previously (Dvoriantchikova et al., 2014). Briefly, macrophages and endothelial cells were removed from the retinal cell suspension by panning with the anti-macrophage antibody (AIA31240, Accurate Chemical, Westbury, NY, USA). The RGCs were then bound to the panning plates containing CD90.2/Thy1.2 hybridoma supernatant and released by trypsin incubation. Notch+ cells (RPCs) and RGCs were used for genomic DNA purification followed by WGBS.

### Whole Genome Bisulfite Sequencing (WGBS)

Genomic DNA was purified using the DNeasy Blood and Tissue Kit (QIAGEN #69504). The DNA concentration of the samples was measured using the Qubit^®^ dsDNA BR Assay Kit (Thermo Fisher Scientific). The DNA quality of the samples was assessed with the Fragment AnalyzerTM and the DNF-487 Standard Sensitivity genomic DNA Analysis Kit (Advanced Analytical). WGBS was conducted by Novogene. Briefly, approximately 5 μg of genomic DNA per sample spiked with lambda DNA was fragmented by sonication to 200–400 bp using Covaris S220, followed by end repair and adenylation. Cytosine-methylated barcodes were ligated to the sonicated DNA, and then these fragments were treated with bisulfite (EZ DNA Methylation-GoldTM Kit, Zymo Research, Irvine, CA, USA). The resulting single-stranded DNA fragments were amplified by PCR using KAPA HiFi HotStart Uracil + ReadyMix (2×). The DNA concentration of the libraries was measured using the Qubit^®^ dsDNA HS Assay Kit (Thermo Fisher Scientific, Waltham, MA, USA). The library profiles were checked using the High Sensitivity DNA chip for the 2100 Bioanalyzer (Agilent). WGBS libraries were sequenced on a HiSeq X-10 platform using 150 bp paired-end sequencing.

### WGBS Data Analysis

To obtain the qualified clean data, the raw reads were trimmed using Trimmomatic, a trimming tool for Illumina NGS data, to filter out the contaminated adapter sequence and low-quality reads. The sequenced reads were controlled for quality of sequencing with the FastQC tool. The cleaned reads were then aligned to the Mus musculus reference genome (Genome Reference Consortium 37, mm10) using bismark (Krueger and Andrews, 2011). The cytosine2coverage and bismark_methylation_extractor modules of bismark were employed to identify the methylation state of all cytosines in a CpG, CHH, or CHG context (for every single mappable read) and to compute the percentage of methylation for each CpG site. The average base coverage of the genome was 17 for E11.5 retinas, 22 for E12.5 retinas, 19 for P0 RPCs, 18 for P3 RPCs, and 19 for RGCs (Supplementary Tables S1, S3). The DNA methylation analysis from the WGBS data was performed using Bioconductor R packages “MethylSeekR” and “methylKit” according to the software manuals (Akalin et al., 2012; Burger et al., 2013). Annotation of identified segments and regions was carried out with the R Bioconductor package “Annotatr” (Cavalcante and Sartor, 2017). The promoter was considered as highly-methylated (hypermethylated) if it was located in a segmentation class 3 or 4 genomic region and unmethylated region (UMR) or low-methylated region (LMR; size >500 bp) were not present in the promoter area. To generate the heat maps, we first collected the mean percent of cytosine methylation in promoters of studied genes using “methylKit” and “MethylSeekR” (Supplementary Tables S1, S3, S4). Since genes may have more than one promoter, we selected the promoter with the lowest mean percent of cytosine methylation, providing that the segment (methylKit) or region (UMR or LMR; MethylSeekR) is larger than 500 bp. The mean percent of cytosine methylation was assigned to each relevant gene and used to generate heat maps using Microsoft Excel 2016.

## Results

**Figure 1:**
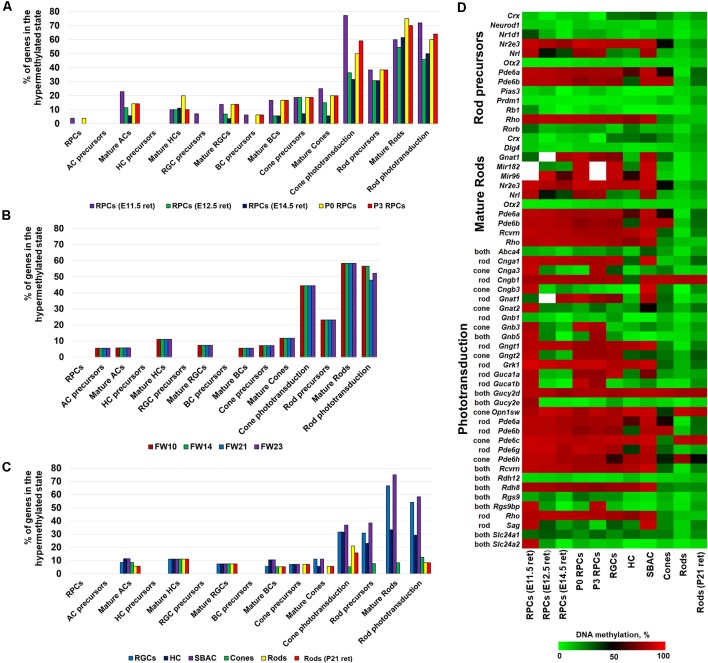
DNA methylation dynamics during retinal progenitor cell (RPC) differentiation into retinal neurons revealed that high methylation levels of photoreceptor phenotypes in the genomic DNA of RPCs (which emerged early in embryogenesis) are inherited by non-photoreceptor retinal neurons, but the DNA methylation levels are sharply reduced in photoreceptor phenotypes. The DNA methylation of non-photoreceptor phenotypes is low in all studied cell types. The % of hypermethylated genes regulating RPC and retinal neuron phenotype development and function in genomes of **(A)** murine retinal progenitors [whole genome bisulfite sequencing (WGBS) data obtained from the current study and NCBI-Gene Expression Omnibus (GEO) WGBS data (GSE87062) for E14.5 retinas] **(B)** human retinal progenitors (NCBI-GEO GSE87061) **(C)** mature retinal neurons [retinal ganglion cell (RGC) WGBS data obtained from the current study and NCBI-GEO WGBS data (GSE84589) for horizontal cell (HC), starburst amacrine cell (SBAC), cones, and rods]. **(D)** The generated heat map reflects DNA methylation dynamics in promoters of photoreceptor genes during RPC differentiation into retinal neurons.

To study the dynamics of DNA methylation in promoters of genes that regulate RPC differentiation into retinal phenotypes and are required for their function in mature states, we first used genomic DNA isolated from embryonic-day (E) 11.5 and 12.5 murine retinas. The majority of cells in the retinas at these time points are RPCs. We also isolated Notch1+ cells (Notch1 is a marker of RPCs) from postnatal-day (P) 0 and 3 retinas using an immunomagnetic cell separation protocol as described previously (Dvoriantchikova et al., 2015). The WGBS libraries, prepared using genomic DNA isolated from these samples, were sequenced and bisulfite sequence reads were then aligned using the Bismark software package. In our analysis, we also included NCBI-GEO WGBS data (GSE87062) for E14.5 retinas (Aldiri et al., 2017). Since WGBS can identify and characterize cytosine methylation in the entire genome with single nucleotide resolution, it allows us to identify differentially methylated regions (methylome states) within the same genome in a more statistically significant manner. To identify the methylome states within the studied genomes, we employed the Bioconductor R packages “methylKit” and “MethylSeekR,” which identify segmentation classes based only on the average DNA methylation level and UMRs or LMR from bisulfite-sequencing data, respectively (Akalin et al., 2012; Burger et al., 2013). The results of the analysis were annotated using the Bioconductor R package “annotatr,” which allowed us to identify gene promoters residing in the identified segments and regions (Cavalcante and Sartor, 2017). Using these datasets, we extracted the methylome data on promoters of key genes required for the development and function of RPCs and retinal neurons. The lists of genes were acquired from LifeMap Discovery and The Stem Cell Research Database. The list of genes required for phototransduction was extracted from the RGD database^1^. All these data were included in Supplementary Table S1. The promoter was considered highly methylated (hypermethylated) if it was located in a segmentation class 3 or 4 genomic region and a UMR or LMR (size >500 bp) was not present in the promoter area. We then calculated the percentage of hypermethylated genes (genes with hypermethylated promoters) relative to the total gene number required for development or function of the studied retinal phenotypes (Figure 1A, Supplementary Table S1). The results of our analysis indicate that promoters of the majority of genes necessary for RPCs and all non-photoreceptor precursor phenotypes were located in unmethylated or low-methylated regions of the genomic DNA of all tested retinal progenitors (RPCs; Figure 1A, Supplementary Table S1). While we found a higher percentage of promoters of genes required for mature non-photoreceptor neurons located in highly methylated regions of the RPC genomes, the majority of these promoters were still unmethylated or low-methylated (Figure 1A, Supplementary Table S1). Opposed to our observations for RPCs and non-photoreceptor retinal phenotypes, we found that many promoters of genes controlling rod photoreceptor development and the rod and cone phototransduction process were located in highly methylated regions of RPC genomic DNA (Figure 1A, Supplementary Table S1). Promoters of many genes critical for photoreceptor development and function were located in genomic regions with a methylation level of 80% or higher (Supplementary Table S1). These genes include *Nr2e3* (which defines the rod phenotype), rod and cone opsins (*Rho* and *Opn1sw*), as well as genes critical for cone and rod phototransduction processes (*Cnga1, Cngb1, Gngt1, Grk1, Pde6a, Pde6b, Pde6c, Pde6h, Pde6g, Rcvrn*, and* Rdh8*; Supplementary Table S1). To test whether observed methylation patterns in RPC genomic DNA are mouse-specific or present in other species, we analyzed WGBS data from human retinas at fetal week (FW) 10, 14, 21, and 23 (NCBI-GEO GSE87061) as described above (Aldiri et al., 2017). The majority of cells in human fetal retinas at these time points are RPCs. We observed similar methylation patterns for retinal phenotype-related genes (Figure 1B, Supplementary Table S2). Importantly, many of the same photoreceptor and phototransduction genes were highly methylated in both species (Supplementary Tables S1, S2).

To analyze DNA methylation dynamics during RPC differentiation into retinal neurons, we then examined the methylation profiles of genomic DNA isolated from murine RGCs, horizontal cells (HC), starburst amacrine cells (SBAC), cone photoreceptors, rod photoreceptors, and the retinas of P21 mice (which contain mostly rod photoreceptors; the adult murine retina is made up of approximately 70% rods). To collect WGBS data, we isolated RGCs from P14 mice using the two-step immunopanning protocol (Dvoriantchikova et al., 2014). Genomic DNA was isolated, processed, and analyzed as described above. To study the methylome of HC, SBAC, cones, and rods we used NCBI-GEO WGBS data (GSE84589; Hartl et al., 2017). In our analysis, we included NCBI-GEO WGBS data (GSE87062) to study the methylome of P21 retinas (Aldiri et al., 2017). We found that the methylomes of RGCs, HC, and SBAC had very similar patterns to RPCs regarding the genes required for retinal phenotype development and function (Figures 1A,C, Supplementary Tables S1, S3). It should be noted that the highly methylated regions of RPC genomic DNA, containing promoters of genes like *Slc17a8* and *Th* for mature amacrine cells, *Gja10* for HCs, and *Sncg* and *Opn4* for RGCs, stayed highly methylated in the respective cell types (Supplementary Tables S1, S3). Our findings also indicate that the majority of highly methylated regions of RPCs containing promoters of photoreceptor genes were inherited by genomic DNA of RGCs, HC, and SBAC (Supplementary Tables S1, S3). These genes include *Nr2e3, Gngt1, Grk1, Pde6a, Pde6c, Rcvrn, Rho, Rdh8*, and *Opn1sw*. At the same time, promoters of these genes were located in unmethylated or low-methylated genomic regions of mature rod (*Nr2e3, Gngt1, Grk1, Pde6a, Rcvrn, Rho*, and* Rdh8*) and cone (*Nr2e3, Gngt1, Grk1, Pde6a, Pde6c, Rcvrn, Rdh8*, and *Opn1sw*) photoreceptors (Figure 1C, Supplementary Table S3) as was expected. The methylome patterns for genes required for non-photoreceptor retinal phenotypes in rod and cone genomes were similar to the methylome patterns in RGC, HC, and SBAC genomes (Figure 1C, Supplementary Table S3). To clearly demonstrate the changes in DNA methylation levels in promoters of photoreceptor genes during RPC differentiation into retinal phenotypes, we generated a heat map showing the mean percent of cytosine methylation in genomic regions containing promoters of target genes in the studied retinal cell types (Figure 1D, Supplementary Table S3). All of these data suggest that RPC differentiation into non-photoreceptor phenotypes mostly excludes the DNA demethylation step (at least regarding the studied genes), while the DNA demethylation step is a part of RPC differentiation into photoreceptors. Our data indicate that the DNA demethylation step mostly affects regions containing genes critical for rod photoreceptor development and rod and cone phototransduction processes (Figure 1).

**Figure 2:**
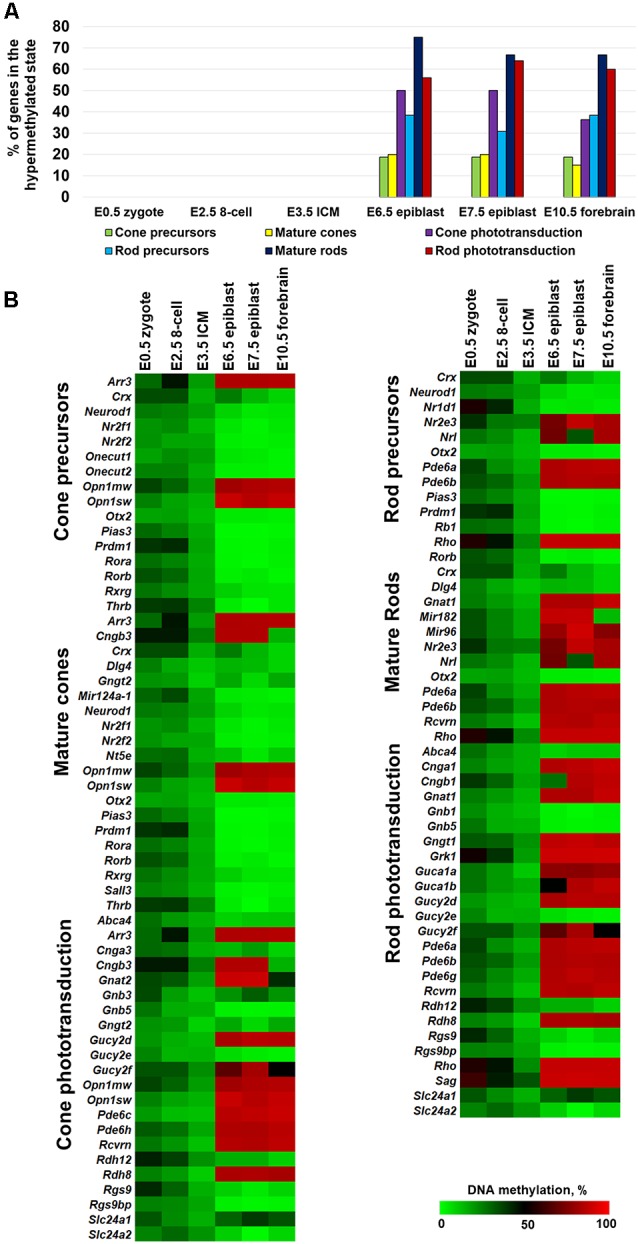
The methylation levels of genomic regions containing promoters of photoreceptor genes were sharply increased during the transition from the inner cell mass stage (ICM; E3.5) to the epiblast stage (E6.5). **(A)** Percentage of hypermethylated genes required for photoreceptor phenotypes in the genomic DNA of zygote, 8-cell blastocyst, ICM, and epiblast. **(B)** Heat maps show changes in DNA methylation levels in genomic regions containing promoters of photoreceptor genes during the early stages of embryogenesis.

**Figure 3:**
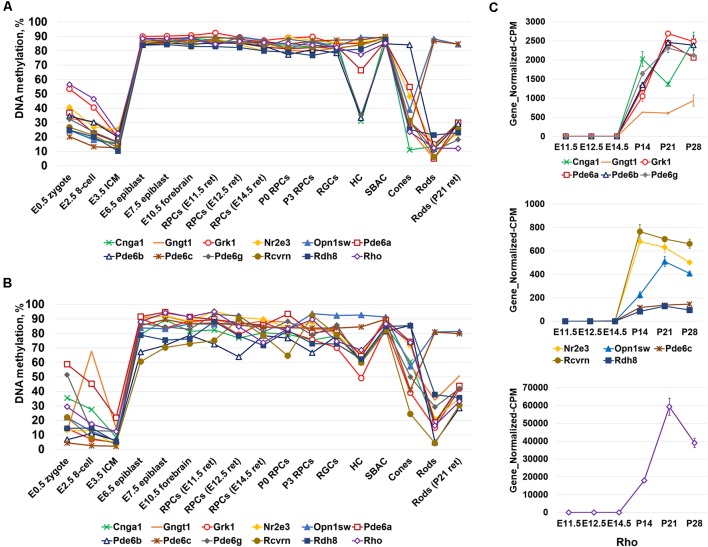
The analysis of DNA methylation in promoters of a subpopulation of photoreceptor and phototransduction genes revealed different dynamics of cytosine methylation in different cell types at different stages of embryogenesis. **(A)** Mean methylation levels were calculated using the “methylKit” and “MethylSeekR” software packages. **(B)** Mean DNA methylation levels based on the methylation of individual CpGs in the promoter and first exon regions of photoreceptor and phototransduction genes were reduced in mature photoreceptors during retinal development. **(C)** Reduced methylation of cytosines in close proximity to a transcription start site (TSS) in photoreceptor genes was accompanied by increased expression of these genes (NCBI-GEO RNA-seq data GSE101986; Gene_Normalized-CPM is the normalized gene level at counts-per-million).

Since photoreceptor phenotypes were highly methylated, while the majority of genes required for non-photoreceptor phenotypes were located in low-methylated or unmethylated regions of genomic DNA of all studied retinal cell types, we questioned at which embryogenesis stage high DNA methylation levels appear in photoreceptor gene-containing regions. To answer the question, we collected WGBS data from NCBI-GEO (GSE98151, GSE84235, GSE101360) for the zygote stage (E0.5), 8-cell stage (E2.5), inner cell mass stage (ICM; E3.5), E6.5 and E7.5 epiblasts, and the E10.5 forebrain (since both the eyes and the forebrain originate from the anterior neural plate; ENCODE Project Consortium, 2012; Smith et al., 2017; Wang et al., 2018). We performed the same analysis with the WGBS datasets as above and found that all genomic regions containing promoters of genes necessary for rod photoreceptor development and rod and cone phototransduction processes were unmethylated or low-methylated until the E3.5 ICM stage (Figure 2A, Supplementary Table S4). Meanwhile, these regions in E6.5 epiblast genomic DNA and later were already highly methylated (Figure 2A, Supplementary Table S4). The heat maps generated using our data perfectly reflect these changes in the DNA methylation levels within genomic regions containing promoters of photoreceptor-related genes (Figure 2B, Supplementary Table S4). Thus, the high DNA methylation levels in photoreceptor gene-containing regions appearing in genomic DNA at early stages of embryogenesis and later were inherited by descendants (Figure 2). These regions are demethylated in photoreceptor phenotypes during RPC differentiation but not in non-photoreceptor retinal phenotypes like RGCs, HC, and SBAC (Figure 1). In the next step, we tested the methylation dynamics in promoters of a group of 12 key genes required for rod and cone photoreceptor development and function. We first collected the mean percent of cytosine methylation in promoters of these genes using methylKit and MethylSeekR calculated data (Figure 3A, Supplementary Table S5). We found that the relatively high (but mostly less than 60%) methylation level at the zygote stage is decreasing to reach values in the range of 10%-27% at the ICM stage. The methylation level in these promoters is sharply increased (80% or more) in DNA of E6.5 epiblasts. These highly methylated regions are inherited further by descendants and demethylated in the DNA of photoreceptors (Figure 3A, Supplementary Table S5). Since the methylKit and MethylSeekR R Bioconductor packages provide only the mean percent of methylation per genomic region, we collected data of the methylation % of individual cytosine bases in the promoter region and first exon of studied genes and calculated the mean % of DNA methylation using these datasets (Figure 3B, Supplementary Table S5). We found that the methylation dynamics for these genes were similar to the above case (Figures 3A,B). Our data indicate that the highly methylated cytosines (80% or more) were mostly located close to the transcription start site (TSS) in many of these genes (Supplementary Table S5). To evaluate whether reduced DNA methylation levels in the 12 studied genes (Figures 3A,B, Supplementary Table S5) was accompanied by increased expression of these genes, we analyzed NCBI-GEO RNA-seq data (Series GSE101986) for E11.5, E12.5, and E14.5 retinas, which mostly contain RPCs, and data for P14, P21, and P28 retinas—while retinas at these time points reflect the rod photoreceptor epigenetic state at the DNA level, all highly expressed genes related to all retinal phenotypes can be detected in these retinas at the RNA level (Hoshino et al., 2017). As expected, we found that retinas with lower methylation levels of the studied photoreceptor genes had higher expression of these genes (Figure 3C, Supplementary Table S5).

## Discussion

Over the past 20 years, we have achieved considerable clarity in our understanding of signaling cascades that control retinal development (Xiang, 2013). However, our knowledge of the epigenetic mechanisms in retinal development is still limited (Corso-Díaz et al., 2018). In order to fill this gap, we studied DNA methylation (the epigenetic mechanism used by cells to control gene expression) during retinal development. We investigated the dynamics of genomic DNA methylation in order to evaluate the role of the DNA methylation and DNA demethylation pathways during RPC differentiation into retinal neurons. Our analysis of DNA methylation in RPCs and retinal neurons suggested the important role of the DNA demethylation pathway for rod photoreceptor development and for rod and cone phototransduction processes. At the same time, our data suggest that the DNA methylation and DNA demethylation pathways may be less important for the development and function of non-photoreceptor phenotypes.

The generally accepted epigenetic model for differentiation of stem cells or progenitors into a particular cell type states that DNA methylation silences gene expression required for other phenotypes, allowing only the expression of genes required for this particular phenotype (Corso-Díaz et al., 2018). DNA methylation also prevents regression of differentiated cells into an undifferentiated state. The role of the DNA methylation pathway is critical in this model: Dnmt3a and Dnmt3b methylate and prevent activation of unnecessary genes, while Dnmt1 preserves the phenotype in descendants (Corso-Díaz et al., 2018). However, our data indicate that promoters of genes for non-photoreceptor retinal phenotypes were located in unmethylated or low-methylated regions of RPC genomic DNA and they remain unmethylated or low-methylated in all studied retinal phenotypes. Thus, Dnmt3a and Dnmt3b activity (at least regarding the tested genes) may not be required for RPC differentiation into non-photoreceptor phenotypes. Dnmt1 activity is still necessary to preserve DNA methylation in RPC descendants. Meanwhile, we found that promoters of photoreceptor genes belong to highly methylated regions of RPC genomic DNA. These regions were low-methylated or unmethylated in mature photoreceptors followed by increased expression of these genes. DNA demethylation can be achieved passively due to cell proliferation combined with reduced Dnmt1 activity in the proliferating cells (Koh and Rao, 2013; Huang and Rao, 2014). However, RPCs are the only cells in developing retina that can proliferate. Hence, a passive DNA demethylation process should significantly reduce methylation of RPC genomic regions containing photoreceptor genes. Our data suggest that this is not the case and an active DNA demethylation process is required to demethylate these genomic regions in RPCs to allow photoreceptor phenotypes during retinal development. Thus, the DNA demethylation pathway powered by TET demethylase activity, but not the DNA methylation pathway, might be critical for photoreceptor development. Combining the data together, we can suggest that the roles of Dnmt3a and Dnmt3b in the differentiation of retinal neurons are not important. Normal retinal development in double Dnmt3a/Dnmt3b knockout mice supports this conclusion (Singh et al., 2017). However, Dnmt1 activity may be critical to preserving retinal phenotypes during development. The active DNA demethylation pathway might not be directly important for non-photoreceptor phenotypes, but may likely be critical for photoreceptor differentiation. Impaired photoreceptor development in TET demethylase knockouts supports this conclusion (Seritrakul and Gross, 2017). The same publication showed abnormalities in RGC development in TET knockouts. In our study, promoters of the majority of genes required for RGC development and function, including Pou4f2 (Brn3b) and Isl1, were located in unmethylated or low-methylated regions of RPC and RGC genomes, suggesting the role of other TET-regulated signaling cascades in RGC development. Seritrakul and Gross demonstrated that inhibition of the Wnt and Notch pathways in developing zebrafish Tet2/Tet3 knockout retinas restores the ability of RPCs to differentiate into RGCs, but not into photoreceptors, the majority of which remained undeveloped in these animals (Seritrakul and Gross, 2017). This data suggests that TET demethylases might facilitate demethylation and transcriptional activation of the genes coding inhibitors of these pathways (which have not been identified yet) promoting RGC differentiation. Meanwhile, highly methylated promoters of genes required for photoreceptor development and function still require TET demethylase activity, and inhibition of the Wnt and Notch pathways is not enough to promote photoreceptor phenotypes in the retinas of these Tet2/Tet3 deficient animals. Thus, the DNA demethylation pathway powered by TET demethylase activity might directly regulate photoreceptor phenotypes and indirectly regulate the development of different retinal phenotypes (for example, RGCs due to Wnt and Notch pathways).

During the early embryonic stages (from zygote to blastocyst) genomic DNA undergoes massive and global DNA demethylation that leads to the lowest genomic DNA methylation levels in the 32–64 cell blastocyst (Messerschmidt et al., 2014). Starting from this moment, the global DNA methylation level only increases (Messerschmidt et al., 2014). Our data suggest that DNA methylation in genomic regions containing photoreceptor genes appear as early as the E6.5 epiblast stage and remains until the RPC stage. We can speculate that Dnmt3a and Dnmt3b activity in these regions is directed at genes that must be inactivated to allow the epiblast phenotype, also affecting methylation of rod photoreceptor and rod and cone phototransduction genes. Meanwhile, at the RPC stage, the demethylation of these genomic regions is required to allow photoreceptor phenotypes, but is not required for RPC differentiation into non-photoreceptor retinal neurons. The analysis of the methylation dynamics of individual cytosine bases in the promoter and first exon regions of photoreceptor genes revealed that the cytosines close to a TSS were mostly affected. We found that low methylation levels of these cytosines at the ICM (E3.5) stage were sharply increased at the epiblast stage. Later, when RPCs start to differentiate into photoreceptors, the methylation of these cytosines was reduced, followed by increased expression of photoreceptor genes (Supplementary Table S5). These data suggest that methylation of these cytosines may affect the initiation of transcription of the studied genes. The role of methylated cytosine bases in close proximity to a TSS in silencing gene expression was shown previously (Corso-Díaz et al., 2018). We cannot exclude the possibility that methylation of enhancers of photoreceptor genes might affect the expression of these genes. However, our limited knowledge of known enhancers in genomic regions containing photoreceptor genes restricted our ability to investigate the methylation levels of these regulatory elements. To sum up our results, RPC genomic DNA contains genes of non-photoreceptor phenotypes in unmethylated or low-methylated regions. Our findings suggest that no methylation is required to inactivate unnecessary phenotypes during RPC differentiation into retinal phenotypes (i.e., for example, RPC differentiation into RGCs is not accompanied by methylation of genes required for HC, SBAC, rod, or cone phenotypes). Thus, our data and published literature suggest that the DNA methylation pathway is not critical for retinal development (Dnmt3a and Dnmt3b in particular; however, Dnmt1 is still required to preserve methylation patterns during RPC proliferation). At the same time, activity of the DNA demethylation pathway is critical for the generation of functional photoreceptors.

## Data Availability

The datasets generated and analyzed during the current study are available in the NCBI Gene Expression Omnibus GEO database (accession number: GSE126474). In addition, other NCBI-GEO data used in the study can be accessed with the following GEO accession numbers: GSE87062, GSE84589, GSE98151, GSE84235, GSE101360, and GSE101986.

## Ethics Statement

All experiments were performed in compliance with the National Institutes of Health (NIH) Guide for the Care and Use of Laboratory Animals, the Association for Research in Vision and Ophthalmology (ARVO) statement for use of animals in ophthalmic and vision research, and the University of Miami Institutional Animal Care and Use Committee’s (IACUC) approved protocol.

## Author Contributions

GD purified the RGCs, RPCs, and carried out the experiments. GD and RS assisted with the WGBS. DI and RS assisted with the bioinformatic analysis. DI conceived and supervised the project, analyzed the data, and wrote the manuscript. All authors read and approved the final manuscript.

## Conflict of Interest Statement

The authors declare that the research was conducted in the absence of any commercial or financial relationships that could be construed as a potential conflict of interest.

## Footnotes

^1^https://rgd.mcw.edu/wg/

## Supplementary Material

TABLE S1 Methylome states of promoters of genes necessary for RPC and retinal neuron phenotypes in genomic DNA of murine retinal progenitors (RPCs). All highly-methylated genes are labeled in orange bold/italic.

TABLE S2 Methylome states of promoters of genes necessary for RPC and retinal neuron phenotypes in genomic DNA isolated from human fetal retinas. All highly-methylated genes are labeled in orange bold/italic.

TABLE S3 Methylome states of promoters of genes necessary for RPC and retinal neuron phenotypes in genomic DNA of murine RGCs, HC, SBAC, cones, and rods. All highly-methylated genes are labeled in orange bold/italic.

TABLE S4 Methylome states of promoters of genes necessary for photoreceptor phenotypes in genomic DNA isolated from the zygote (E0.5), 8-cell stage embryo (E2.5), the inner cell mass (ICM; E3.5), E6.5 and E7.5 epiblasts and the E10.5 forebrain. All highly-methylated genes are labeled in orange bold/italic.

TABLE S5 Mean methylation levels in promoter regions of photoreceptor genes calculated using the methylKit and MethylSeekR software packages. Changes in mean methylation levels are based on the methylation of individual CpGs in the promoter and first exon regions of photoreceptor genes during embryogenesis and retinal development.

## References

[B1] Akalin A. Kormaksson M. Li S. Garrett-Bakelman F. E. Figueroa M. E. Melnick A.. (2012). methylKit: a comprehensive R package for the analysis of genome-wide DNA methylation profiles. Genome Biol. 13:R87. 10.1186/gb-2012-13-10-r87 23034086 PMC3491415

[B2] Aldiri I. Xu B. Wang L. Chen X. Hiler D. Griffiths L.. (2017). The dynamic epigenetic landscape of the retina during development, reprogramming, and tumorigenesis. Neuron 94, 550.e10–568.e10. 10.1016/j.neuron.2017.04.022 28472656 PMC5508517

[B3] Burger L. Gaidatzis D. Schübeler D. Stadler M. B. (2013). Identification of active regulatory regions from DNA methylation data. Nucleic Acids Res. 41:e155. 10.1093/nar/gkt599 23828043 PMC3763559

[B4] Cavalcante R. G. Sartor M. A. (2017). annotatr: genomic regions in context. Bioinformatics 33, 2381–2383. 10.1093/bioinformatics/btx183 28369316 PMC5860117

[B6] Corso-Díaz X. Jaeger C. Chaitankar V. Swaroop A. (2018). Epigenetic control of gene regulation during development and disease: a view from the retina. Prog. Retin. Eye Res. 65, 1–27. 10.1016/j.preteyeres.2018.03.002 29544768 PMC6054546

[B7] Dvoriantchikova G. Perea-Martinez I. Pappas S. Barry A. F. Danek D. Dvoriantchikova X.. (2015). Molecular characterization of notch1 positive progenitor cells in the developing retina. PLoS One 10:e0131054. 10.1371/journal.pone.0131054 26091508 PMC4474692

[B8] Dvoriantchikova G. Santos A. R. Saeed A. M. Dvoriantchikova X. Ivanov D. (2014). Putative role of protein kinase C in neurotoxic inflammation mediated by extracellular heat shock protein 70 after ischemia-reperfusion. J. Neuroinflammation 11:81. 10.1186/1742-2094-11-81 24755298 PMC4001362

[B5] ENCODE Project Consortium. (2012). An integrated encyclopedia of DNA elements in the human genome. Nature 489, 57–74. 10.1038/nature11247 22955616 PMC3439153

[B9] Hartl D. Krebs A. R. Juttner J. Roska B. Schubeler D. (2017). Cis-regulatory landscapes of four cell types of the retina. Nucleic Acids Res. 45, 11607–11621. 10.1093/nar/gkx923 29059322 PMC5714137

[B10] Hoshino A. Ratnapriya R. Brooks M. J. Chaitankar V. Wilken M. S. Zhang C.. (2017). Molecular anatomy of the developing human retina. Dev. Cell 43, 763.e4–779.e4. 10.1016/j.devcel.2017.10.029 29233477 PMC5776731

[B11] Huang Y. Rao A. (2014). Connections between TET proteins and aberrant DNA modification in cancer. Trends Genet. 30, 464–474. 10.1016/j.tig.2014.07.005 25132561 PMC4337960

[B12] Kim J. W. Yang H. J. Brooks M. J. Zelinger L. Karakulah G. Gotoh N.. (2016). NRL-regulated transcriptome dynamics of developing rod photoreceptors. Cell Rep. 17, 2460–2473. 10.1016/j.celrep.2016.10.074 27880916 PMC5131731

[B13] Koh K. P. Rao A. (2013). DNA methylation and methylcytosine oxidation in cell fate decisions. Curr. Opin. Cell Biol. 25, 152–161. 10.1016/j.ceb.2013.02.014 23498662 PMC3649866

[B14] Krueger F. Andrews S. R. (2011). Bismark: a flexible aligner and methylation caller for Bisulfite-Seq applications. Bioinformatics 27, 1571–1572. 10.1093/bioinformatics/btr167 21493656 PMC3102221

[B15] Messerschmidt D. M. Knowles B. B. Solter D. (2014). DNA methylation dynamics during epigenetic reprogramming in the germline and preimplantation embryos. Genes Dev. 28, 812–828. 10.1101/gad.234294.113 24736841 PMC4003274

[B16] Perera A. Eisen D. Wagner M. Laube S. K. Künzel A. F. Koch S.. (2015). TET3 is recruited by REST for context-specific hydroxymethylation and induction of gene expression. Cell Rep. 11, 283–294. 10.1016/j.celrep.2015.03.020 25843715

[B17] Seritrakul P. Gross J. M. (2017). Tet-mediated DNA hydroxymethylation regulates retinal neurogenesis by modulating cell-extrinsic signaling pathways. PLoS Genet. 13:e1006987. 10.1371/journal.pgen.1006987 28926578 PMC5621703

[B18] Singh R. K. Mallela R. K. Hayes A. Dunham N. R. Hedden M. E. Enke R. A.. (2017). Dnmt1, Dnmt3a and Dnmt3b cooperate in photoreceptor and outer plexiform layer development in the mammalian retina. Exp. Eye Res. 159, 132–146. 10.1016/j.exer.2016.11.014 27865785

[B19] Smith Z. D. Shi J. Gu H. Donaghey J. Clement K. Cacchiarelli D.. (2017). Epigenetic restriction of extraembryonic lineages mirrors the somatic transition to cancer. Nature 549, 543–547. 10.1038/nature23891 28959968 PMC5789792

[B20] Wang C. Liu X. Gao Y. Yang L. Li C. Liu W.. (2018). Reprogramming of H3K9me3-dependent heterochromatin during mammalian embryo development. Nat. Cell Biol. 20, 620–631. 10.1038/s41556-018-0093-4 29686265

[B21] Xiang M. (2013). Intrinsic control of mammalian retinogenesis. Cell. Mol. Life Sci. 70, 2519–2532. 10.1007/s00018-012-1183-2 23064704 PMC3566347

[B22] Xu Y. Xu C. Kato A. Tempel W. Abreu J. G. Bian C.. (2012). Tet3 CXXC domain and dioxygenase activity cooperatively regulate key genes for Xenopus eye and neural development. Cell 151, 1200–1213. 10.1016/j.cell.2012.11.014 23217707 PMC3705565

